# *Plasmodium knowlesi* – an emerging pathogen

**DOI:** 10.1111/voxs.12115

**Published:** 2015-04-13

**Authors:** M A Ahmed, J Cox-Singh

**Affiliations:** 1 School of Medicine, University of St AndrewsSt Andrews, UK; 2 Department of Parasitology, Faculty of Medicine, University of MalayaKuala Lumpur, Malaysia

**Keywords:** Plasmodium, knowlesi, malaria, pathophysiology, virulence, zoonosis

## Abstract

Ten years have passed since the publication of a large focus of *Plasmodium knowlesi* infections in the human population. The discovery was made during a molecular investigation of atypical *P. malariae* cases in the Kapit Health Division, Sarawak, Malaysian Borneo. Patients were more symptomatic with higher parasite counts than expected in *P. malariae* infections. The investigation found only *P. knowlesi* DNA present in patient blood samples. Morphological similarity had allowed *P. knowlesi* to masquerade as *P. malariae* during routine diagnostic microscopy for malaria. *P. knowlesi*, a malaria parasite of macaque monkeys, had entered the human population. The subsequent development of *P. knowlesi* species-specific PCR assays soon demonstrated that the entry was not confined to the Kapit Division but extended across island and mainland Southeast Asia. Relevant clinical descriptions and guidelines for the treatment and management of patents with *P. knowlesi* malaria were not available. Nor was it clear whether *P. knowlesi* had undergone a host switch event into the human population or if infections were zoonotic. The outputs of studies on *P. knowlesi* malaria during the past 10 years will be summarized, highlighting major findings within the context of pathophysiology, virulence, host switch events, treatment, control and importantly malaria elimination.

## Introduction

Malaria parasites, member species of the genus *Plasmodium*, are blood-borne and pose a substantial risk in blood transfusion related infection [1–5]. Donor blood infected with any of the human-host-adapted *Plasmodium* species, *P. falciparum*, *P. vivax*, *P. ovale* and *P. malariae* or newly emergent species such as *P. knowlesi* would cause malaria in the untreated non-immune recipient. Most medical texts and guidelines for malaria provide detailed information on the human–host-adapted malaria parasites but not on emergent species. *P. knowlesi*, a parasite of the long and pig-tailed macaques of Southeast Asia, is not vertebrate host restricted, and humans, under experimental conditions, were permissive to infection [6]. Clinical information collected from therapeutically induced *P. knowlesi* infections in patients with tertiary syphilis provides information on human–parasite interaction using blood passage of laboratory lines of *P. knowlesi* [6]. However, naturally acquired *P. knowlesi* malaria was not recognized and therefore clinically overlooked, until a large focus of *P. knowlesi* was described in the human population in Sarawak, Malaysian Borneo, in 2004 [7]. *P. knowlesi* is morphologically indistinguishable from *P. malariae*, one of the more benign human-host-adapted *Plasmodium* species, and infections were misdiagnosed until the recent development of *P. knowlesi*-specific PCR-based assays [7–9]. The use of these assays indicate that zoonotic malaria, caused by *P. knowlesi,* is widespread in Southeast Asia and, in stark contrast to *P. malariae,* is not benign [10–13].

## *Plasmodium knowlesi* pathophysiology

**Fig 1:**
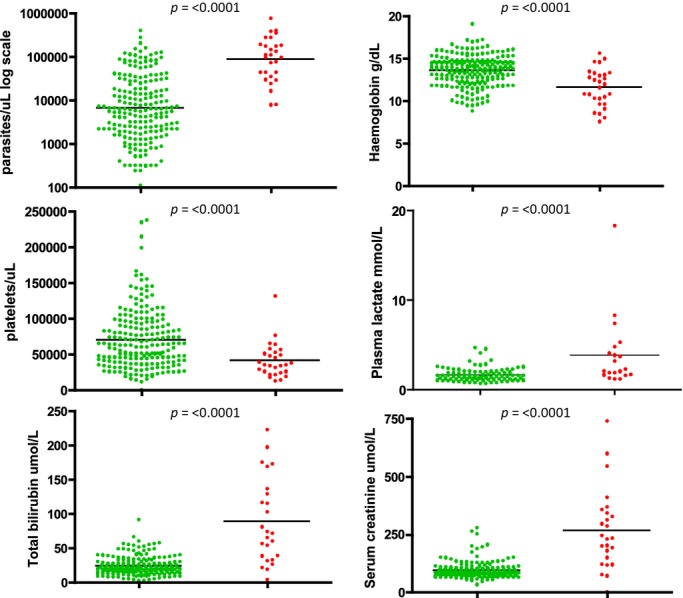
Complicated vs. uncomplicated *Plasmodium knowlesi* disease groups. Patients with *P. knowlesi* malaria grouped by uncomplicated disease [U = Green] and complicated disease [C = Red]. Plasma lactate results from a subset of patients (U = 91, C = 20, *n* = 111). P values were calculated using the Mann–Whitney *U*-test for nonparametric data and the unpaired *t*-test for normally distributed data (Prism 4 for Macintosh; GraphPad Software, Inc. San Diego).

Serial blood passage of a limited number of experimental lines of *P. knowlesi* in neurosyphilis patients, pre-antibiotic pyretic treatment in the 1950s, was associated with increased parasite virulence [6]. The next account of *P. knowlesi* pathophysiology came with the first documented case of naturally acquired *P. knowlesi* published in 1965 [14]. The case was diagnosed retrospectively and, while the patient was symptomatic, he recovered fully following treatment and no other cases were confirmed. In fact, when we published the large focus of *P. knowlesi* in patients with single-specie*s P. knowlesi* infections in Kapit, Sarawak, we stated that *P. knowlesi* caused the usual symptoms of malaria; all patients responded to treatment and no deaths were reported [7]. This changed during a subsequent study when four P. knowlesi deaths were confirmed retrospectively. Only one of these was reported as a malaria death, P. falciparum, the others were attributed to other causes [11]. There was no information on severe knowlesi malaria in the literature or medical texts to guide healthcare professionals towards correct diagnosis and treatment of these patients. *P. knowlesi* malaria was new to the medical field, clinicians and other healthcare professionals were unaware that they were dealing with a newly described virulent form of malaria that, in practice, was most often misdiagnosed as the more benign *P. malariae*. A prospective clinical study on PCR-confirmed single-species *P. knowlesi* infected patients confirmed the need for knowlesi-specific treatment and management guidelines [12]. In that study 107 patients had *P. knowlesi* malaria, ten had complications and two died [12]. Complications included >100 000 parasites/*μ*l blood, acute renal impairment, jaundice, hypotension, lactate acidosis, acute and late onset respiratory distress syndrome. All of these complications are included in the WHO guidelines for severe falciparum malaria in the non-immune [15]. Notably in this and subsequent studies, severe malaria with coma was not described in severe *P. knowlesi* infections [11,12,16,17]. Severe malarial anaemia was reported in children but not adults and was not as severe as in the *P. falciparum* comparator group [18]. In a recent study, 232 adult patients with PCR-confirmed single-species *P. knowlesi* infections were recruited in Sarawak Malaysian Borneo [19]. Twenty-eight (12%) of these had complications and four (1·7%) patients died, Table 1. Parasitaemia, total bilirubin, serum creatinine and plasma lactate were significantly higher in the complicated group, Fig.1. Parasitaemia was not used as a criterion to score severe disease. The study of *P. knowlesi* malaria is relatively new, and markers of severe disease are preliminary but suggest that patients with parasitaemia >35 000 parasites/*μ*l, bilirubin > 43 *μ*mol/l, serum creatinine >256 *μ*mol/l or platelets <45 000/*μ*l are at risk [12,19]. *P. knowlesi* is unusual among the parasites that infect human and non-human primates because the asexual part of the life cycle, the erythrocytic cycle associated with clinical signs and symptoms, takes 24 h to complete rather than 48 and 72 h in other types of malaria. Therefore, parasitaemia increases daily in *P. knowlesi* infections, increased parasitaemia is associated with disease severity, and while multiplication rates are probably variant specific and the number of merozoites produced will need to be factored in, patients with suspected *P. knowlesi* infection require urgent diagnosis and rapid access to optimal treatment.

**Table 1:** Summary of patients with complicated *Plasmodium knowlesi* malaria (*n* = 28)

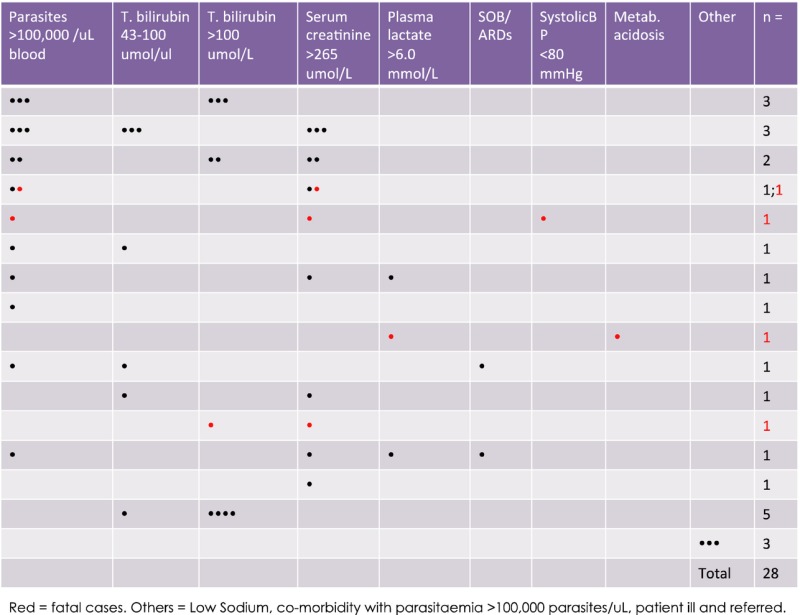

## Virulence

**Fig 2:**
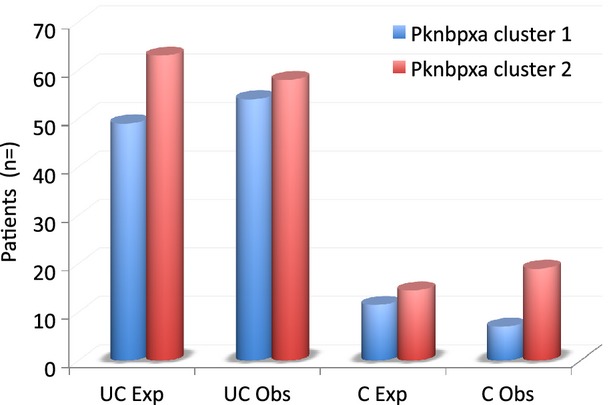
The number of expected and observed cases of *Plasmodium knowlesi* malaria infected with parasites with *Plasmodium knowlesi normocyte binding protein (pknbp) xa* cluster 1 or cluster 2 parasites. *Pknbpxa* haplotypes were generated for 138 patient isolates in a subset of 147 patients. In the study, 44% of patients were infected with *Pknbpxa* cluster 1 type parasites and 56% with *Pknbpxa* cluster 2 type parasites. The patients were then grouped into uncomplicated (UC) *n* = 112 and complicated (C) *n* = 28 groups. The expected (Exp) number of patients in the UC and C groups was calculated based on the 44:56 ration of cluster 1 to cluster infections and compared to the number observed (Obs). More patients with complicated disease (C Obs) were infected with *Pknbpxa* cluster 2 parasites. Chi-square *P* = 0·048; with Yates correction *P* = 0·08 (http://vassarstats.net/tab2x2.html).

*Plasmodium knowlesi* isolates collected from human infections are genetically diverse [7,9]. Parasitaemia is associated with disease severity and we asked the question are some *P. knowlesi* variants more virulent than others? We sequenced two *P. knowlesi* genes responsible for parasite invasion of erythrocytes and found two genetically distinct clusters of one of the *P. knowlesi* genes encoding erythrocyte invasion proteins, *P. knowlesi normocyte binding protein xa* (*Pknbpxa*) [19, 21]. We found that 44% and 56% of patients were infected with *Pknbpxa* cluster 1 and *Pknbpxa* cluster 2, respectively. The clusters contained single nucleotide polymorphisms that allowed for differentiation of haplotype groups with 2 or three alleles among the *P. knowlesi* isolates. Analysis of the alleles with the clinical and laboratory variables in the patient cohort found that patients infected with particular allelic forms of cluster 2 had increased markers of disease severity including parasitaemia [19]. Not all patients with complications were infected with cluster 2 parasites but there were more patients than expected with complications in this cluster (Fig.2). This suggests that, in nature, some *P. knowlesi* variants may be more virulent in the human-host population than others. This study was conducted in Sarawak, Malaysian Borneo, and may explain some of the apparent differential susceptibility of humans to *P. knowlesi* infection in Malaysia and other areas within the region. More work is required to determine genetic diversity of *P. knowlesi* across the Southeast Asian region and associated parasite virulence.

## Host switch events

*Plasmodium knowlesi* infections occur throughout the Southeast Asian region. The region is separated by high mountain ranges and large expanses of water including the South China Sea, the Sulu Sea and Andaman Sea [21]. *P. knowlesi* is a parasite of non-human primates that do not cross oceans and rural human populations at risk tend to be local. It seems likely that, following the primary emergence of *P. knowlesi* and ancient dispersal events, *P. knowlesi,* populations have evolved in local geographical isolation. Each location reporting human infections is most likely dealing with different genetic variants of *P. knowlesl* [22]. The recent discovery of *P. knowlesi* malaria in humans was in Kapit, Malaysian Borneo, and further study found that naturally acquired *P. knowlesi* infections occurred throughout Malaysian Borneo, Peninsular Malaysia, all geographically distinct areas, and subsequently in most parts of Southeast Asia [7,11,21,23]. Therefore, it is important not to extrapolate studies on genetic diversity and virulence between geographically distinct transmission sites.

Even so the multiple entry events recorded across Southeast Asia may have been the result of parasite adaptation to the human host and clonal expansion but the infections are not clonal or related to any one index case or location [7]. A study on *P. knowlesi* isolated from the natural macaque hosts and human infections in Sarawak did not identify genetic clustering of parasites to either host, strongly suggesting a zoonotic origin for the infections in Sarawak [9]. However, children in Sabah were infected without any obvious risk behaviour for acquiring a zoonotic infection, such as spending time in the forest [23]. Also, and perhaps most worrying, *Anopheles balabacensis* is a vector of *P. knowlesi* in Sabah [16]. *An balabacensis* is better known as a vector of *P. falciparum* in Sabah and therefore well placed to channel a host switch event for *P. knowlesi* from macaque-to-human to human-to-human malaria transmission at this location [24,25]. In Vietnam, another geographically distant and distinct location, children have asymptomatic low-level *P. knowlesi* infections mixed with *P. falciparum* and *P. vivax* [26]. *Anopheles dirus* is the main malaria vector in this region of Vietnam and transmits all three parasite species, *P. knowlesi*, *P. falciparum* and *P. vivax,* concurrently [26,27]. *P. knowlesi* warrants careful observation to pre-empt or at least contain a host switch event in any number of Southeast Asian locations – quite a monumental task.

## Treatment

Earlier studies reported that *P. knowlesi* responds well to currently available antimalarial compounds including chloroquine [7,12,28]. The level of sensitivity supports an absence of drug selection pressure implying a recent emergence or zoonotic origin for *P. knowlesi.* Studies designed to properly assess clinical and parasitological responses of *P. knowlesi* to treatment highlight some important differences in response. In 2005, inherent mefloquine resistance of *P. knowlesi* in experimentally infected Rhesus macaques was reported [29]. Also, *P. knowlesi* isolates from human infections responded poorly to mefloquine *ex vivo* [30]. Furthermore, the parasitaemia in a patient, who presented with severe *P. knowlesi* malaria during a second infection, increased following mefloquine treatment [31]. Nonetheless, other patients with uncomplicated *P. knowlesi* infections responded to mefloquine treatment [32,33].

*Plasmodium knowlesi* responds to chloroquine albeit with a higher IC_50_ *ex vivo* [30]. Chloroquine should be used with caution because in the absence of species-specific diagnostic tests in most regions, the risk of misdiagnosing *P. falciparum*, mostly chloroquine resistant, as *P. knowlesi* would put patients at risk.

The most compelling evidence supports the use of artemisinin derivatives either as intravenous artesunate or as oral combination therapies to treat complicated and uncomplicated *P. knowlesi* infections. Patients in Sabah with severe knowlesi malaria were more likely to survive when given intravenous artesunate than quinine [28]. In another study in Sabah patients with all cause, malaria were given oral artemisinin combination therapy or artesunate IV immediately on diagnosis. Most of the patients had knowlesi malaria, 39% had severe symptoms and all survived [16]. Both studies report shorter parasite clearance times for the artemisinin based therapies than chloroquine or quinine. Also *P. knowlesi* responded better to artemisinin derivatives than all other compounds *ex vivo* [30]. In other studies, IV artesunate performed better than quinine in treating severe falciparum malaria [34]. It should be noted that patients with *P. knowlesi* infections can be symptomatic at low parasitaemia, lower than the sensitivity of thick film microscopy [12]. Currently, there is no specific or sensitive diagnostic test for *P. knowlesi* malaria [35].

## Control of zoonotic malaria

Human cases of *P. knowlesi* malaria occur across Southeast Asia [10]. This region is known for human, animal, plant, insect, geographical and geological diversity [36]. Apart from parts of Malaysia and Vietnam, there is little information on *P. knowlesi* transmission, reservoirs of infection, vectors and risk of human infection [27,37,38]. Without this information, it will be difficult to design control measures for zoonotic *P. knowlesi* acquired in the jungle setting, over and beyond use of prophylaxis when spending time in areas where other patients with *P. knowlesi* have been infected. The pragmatic solution would be to encourage location-specific vector, parasite and natural host monitoring and surveillance. These measures are currently costly, require manpower and are logistically difficult. Perhaps a meeting of minds is required to develop alternative sampling and monitoring methods for forest settings that would capture the required data on parasite prevalence, vector capacity, natural hosts but using minimal manpower. Not an easy problem to solve on the backdrop of competing public health problems at each location. In areas where *P. knowlesi* is transmitted in human settlements or even human-to-human, existing malaria control measures would be expected to be effective.

## Malaria elimination

According to the World Health Organization ‘Malaria *elimination* is the interruption of local mosquito-borne malaria transmission, i.e. the reduction to zero of the incidence of malaria infection in a defined geographical area.’ [39]. Malaysia has an excellent record in malaria control; however, Malaysia and other countries in Southeast Asia, pursuing malaria elimination, need to carefully consider *P. knowlesi*. There is some evidence that *P. knowlesi* enters human populations where national human-host-adapted, malaria control initiatives are successful, a good example of this is Malaysia [13,40]. This poses two questions: (i) Will removal of the human-host-adapted species open a new host niche for *P. knowlesi*? or (ii) By removing human host challenge, particularly with *P. vivax*, are humans more susceptible to *P. knowlesi* through loss of cross protection?

Both scenarios are worth taking seriously. The relatively large number of *P. knowlesi* cases was discovered in Malaysia [7]. The study was prompted by the report of atypical cases of *P. malariae* in the area. It is possible that these cases were more visible as cases of *P. falciparum* and *P. vivax* were controlled. Or were there more of these cases as immunity to malaria declined along with *P. vivax* and *P. falciparum*? Returning to *P. knowlesi* induced pyretic therapy in tertiary syphilis. Patients who were previously treated with *P. vivax* were partially resistant to subsequent *P. knowlesi* infection suggesting a degree cross immunity [6].

There is some debate about the prevalence of zoonotic malaria – Is *P. knowlesi* being better recognized because of heightened awareness and PCR identification? If so, then the prevalence of *P. knowlesi* may not have increased, we are simply looking harder, difficult to argue against. However, the number of cases of *P. knowlesi* in Sabah do seem to be increasing [13]. Regardless, awareness brings the opportunity to deal with a large entry and host switch events that might threaten malaria elimination in the area.

If the ultimate goal is malaria eradication, the WHO definition is much more specific ‘Malaria *eradication* is defined as the permanent reduction to zero of the worldwide incidence of malaria infection caused by a particular malaria parasite species.’ [39]. If eradication is the goal, then it would be possible to eradicate *P. falciparum, P. vivax, P. malariae* and *P. ovale* while still tolerating *P. knowlesi* in Southeast Asia.

## Summary

*Plasmodium knowlesi* has entered the human population in Southeast Asia. People who spend time the sylvan transmission sites are at risk. *P. knowlesi* remains largely zoonotic, but transmission near human settlements is suspected in Sabah. *P. knowlesi* is diverse and causes severe disease in at least 10% of those infected in Sabah and Sarawak. Patients with severe and fatal *P. knowlesi* infections fulfil the WHO criteria for severe falciparum malaria but without coma or severe adult anaemia. Healthcare professionals should suspect *P. knowlesi* in patients and blood donors with a history of time spent in the jungles of Southeast Asia. Blood donor screening protocols, to detect malaria exposure, use recombinant *P. falciparum* and *P. vivax* antigen. While there may be some cross reactivity between *P. knowlesi* and the phylogenetically related *P. vivax*, these reagents could not be used reliably to detect antibodies to *P. knowlesi*. At the moment, the risk of donor blood infection with *P. knowlesi* would be expected to be low; however, a history of time spent in the jungles of Southeast Asia may warrant donor referral for a 1-year period.

Importantly, evidence suggests that rapid diagnosis and immediate treatment with artemisinin derivatives is appropriate for this potentially virulent parasite with a short erythrocytic life cycle.
